# Contralateral bridge fixation of freehand minimally invasive pedicle screws combined with unilateral MIS-TLIF vs. open TLIF in the treatment of multi-segmental lumbar degenerative diseases: A five years retrospective study and finite element analysis

**DOI:** 10.3389/fsurg.2022.1049260

**Published:** 2022-11-02

**Authors:** Yingkai Zhang, Tianyao Zhou, Yutong Gu, Wu Che, Liang Zhang, Yichao Wang

**Affiliations:** ^1^ Department of Orthopaedic Surgery, Zhongshan Hospital Fudan University, Shanghai, China; ^2^ Department of Orthopaedic Surgery, Jinshan Hospital of Fudan University, Shanghai, China; ^3^ Shanghai Southwest Spine Surgery Center, Shanghai, China

**Keywords:** lumbar degenerative disease, multi-segment, transforaminal lumbar interbody fusion, pedicle screw fixation, minimally invasive surgery

## Abstract

**Objective:**

To evaluate the efficacy, safety, feasibility and biomechanical stability of contralateral bridge fixation of freehand minimally invasive pedicle screws (Freehand MIPS) combined with unilateral minimally invasive surgery-transforaminal lumbar interbody fusion (MIS-TLIF) (smile-face surgery) and open TLIF for the treatment of multi-segmental lumbar degenerative diseases (LDDs).

**Methods:**

From January 2013 to January 2016, clinical data of multi-segmental (2- or 3-level) LDDs receiving smile-face surgery or open TLIF were retrospectively collected and analyzed. The back and leg pain VAS and ODI were used to assess clinical outcomes preoperatively and postoperatively. The MacNab criteria were used to evaluate the satisfaction of patient. The disc height (DH), lumbar lordosis (LL) and segmental lordosis angle (SLA) were measured before and after surgery. We used patient's CT data to establish the finite element model of smile-face surgery and open TLIF, and analyze biomechanical stability of two methods.

**Results:**

Smile-face surgery group showed shorter operation time, shorter incision, less blood loss, shorter hospital stay than open TLIF (*P* < 0.05). The back VAS in smile-face surgery group was significantly lower than that in open TLIF immediately and 3 months after surgery, and no significant difference was observed 1 year, 2 years and 5 years after surgery. There was no significant difference in the leg pain VAS and ODI between both groups after surgery. No significant difference was observed between two groups in the DH, LL and SLA. At 5-year follow-up, grade I or II fusion was achieved in 99.00% (100/101) segments of smile-face surgery group and 97.67% (84/86) segments of open TLIF group according to Bridwell system. The complication rate of open TLIF was higher than that of smile-face surgery (24.32% vs. 0%, *P* < 0.01). After verification, the established finite element model can accurately simulate the biological structure of lumbar spine and there was no significant difference in biomechanical stability between two methods.

**Conclusions:**

Smile-face surgery has some advantages over open TLIF including smaller aggression, less blood loss, and lower cost, indicating that it is a good choice of treatment for multi-segmental LDDs. Both methods can achieve good biomechanical stability.

## Introduction

Conventional posterior/transforaminal lumbar interbody fusion (PLIF, TLIF) has yielded satisfactory clinical outcomes for lumbar degenerative diseases (LDDs) (1, 2). However, iatrogenic paraspinal muscle injury, posterior tension band disruption, and approach-related complications are a concern (2, 3). In recent years, minimally invasive surgery-transforaminal lumbar interbody fusion (MIS-TLIF) has been widely used in order to improve open TLIF, which can protect the attachment of paraspinal muscles to bone, avoid the disruption of supraspinous and interspinous ligaments, and decreased the bleeding and postoperative pain (4). But most studies of MIS-TLIF have focused on single-level fusion, there are relatively few reports of MIS-TLIF for two or more levels. In this study, contralateral bridge fixation of freehand minimally invasive pedicle screws (Freehand MIPS) (5–10) combined with unilateral MIS-TLIF for bilateral neurological decompression was performed for the treatment of patients with multi-segmental (2- or 3-level) LDDs. The efficacy, safety, and feasibility of this minimally invasive method were compared with those of open TLIF.

## Materials and methods

This study was approved by the Medical Ethics Committee of Zhongshan Hospital Fudan University (B2015–047), and all methods were carried out in accordance with relevant guidelines and regulations. Before the procedure, all patients provided informed consent.

### Patients

From January 2013 to January 2016, clinical data of multi-segmental (2- or 3-level) LDDs (spondylolisthesis, disc herniation with instability or spinal canal stenosis) who received contralateral bridge Freehand MIPS combined with unilateral MIS-TLIF and open TLIF were retrospectively collected and analyzed.

**Figure 1:**
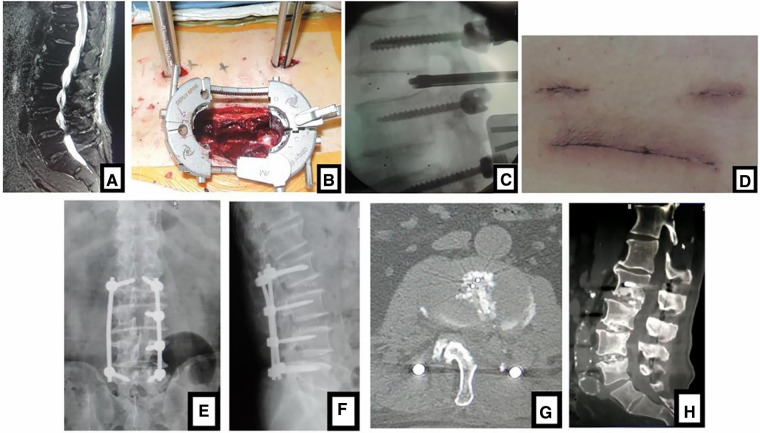
(**A**) sagittal MR images showed 3-level LDD (L2–L5) in a 75-year-old man with neurologic symptoms. (**B**) Contralateral bridge Freehand MIPS combined with unilateral MIS-TLIF through tube was performed. After the cage was inserted into the intervertebral space through the tube, (**C**) the fluoroscopic image confirmed the position of cage. (**D**) The picture was postoperative incision like smile face, so this MIS-TLIF is called smile-face surgery. (**E, F**) Postoperative x-ray showed that the position of pedicle screws and cages was good and (**G**) axial CT scan confirmed that the neurologic decompression was complete. In (**H**) sagittal CT at 5-year follow-up, fusion of grade I or II was achieved.

**Figure 2:**
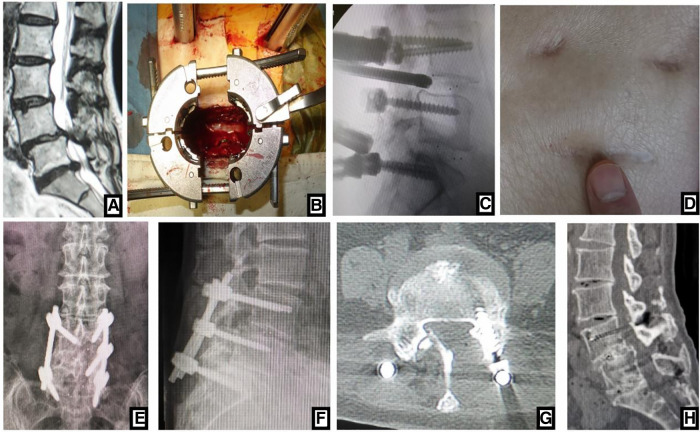
(**A**) sagittal MRI showed 2-level LDD (L3–L5) in a 72-year-old woman with neurologic symptoms. (**B**) Contralateral bridge Freehand MIPS combined with unilateral MIS-TLIF through tube was performed. After the cage was inserted into the intervertebral space through the tube, (**C**) the fluoroscopic image confirmed the position of cage. (**D**) The picture showed the incision like smile face, so this MIS-TLIF is called smile-face surgery. On (**E, F**) postoperative x-ray the position of pedicle screws and cages was good and (**G**) axial CT scan confirmed that the neurologic decompression was complete. On (**H**) sagittal CT at 5-year follow-up, fusion of grade I was achieved.

**Figure 3:**
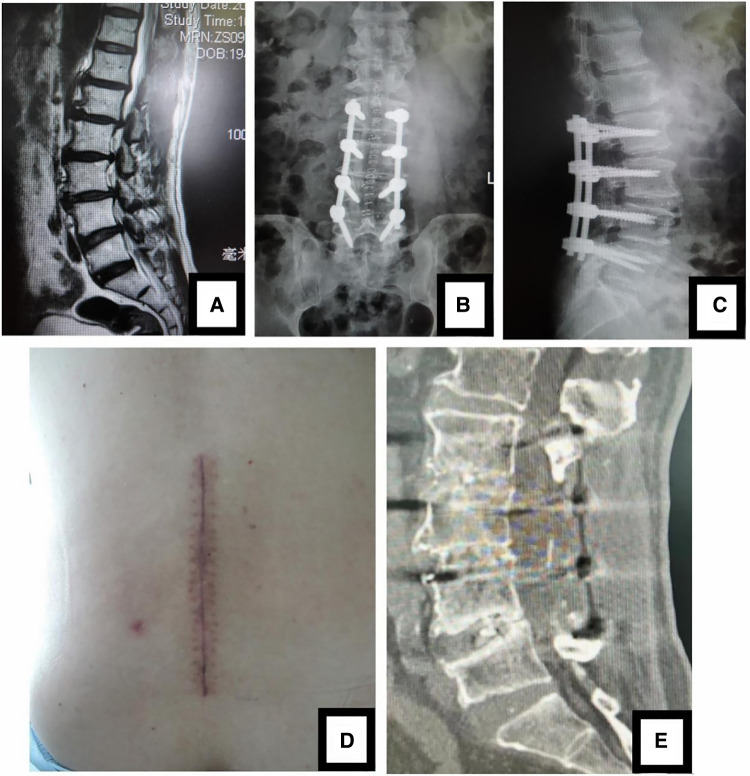
(**A**) sagittal MR images showed 3-level LDD (L2-L5) in a 69-year-old woman. Open TLIF was performed and (**B, C**) postoperative x-ray confirmed that the position of pedicle screws and cages was good. (**D**) The picture was postoperative incision. (**E**) CT scan image at 5-year follow-up showed that the neurologic decompression was complete and fusion of grade I or II was achieved.

The inclusion criteria included: (1) low back pain and leg pain lasting at least 6 months; (2) multi-segmental (2- or 3-level) LDDs of disc herniation with instability, spondylolisthesis (≤ grade II), or lumbar canal stenosis, corresponding to neurological symptoms (Figures 1A, 2A, 3A); (3) no relief of symptoms after conservative treatments and a significant decline in quality of daily life.

The exclusion criteria were as followed: (1) patients with a history of spinal surgery, active infections, lumbar fractures, spine tumors, severe osteoporosis, or severe obesity; (2) patients with coronal and/or sagittal deformities that require surgical correction; (3) any serious psychological problem; (4) degenerative spondylolisthesis with severe instability or isthmic spondylolisthesis.

### Surgical procedure

#### Contralateral bridge freehand MIPS combined with unilateral MIS-TLIF

After general anesthesia, the patient was placed on a radiolucent operating table in a prone position. The pedicles of two end vertebrae involved were identified under fluoroscopy and the skin was marked.

First, Freehand MIPS was performed for the contralateral two end vertebrae (5–10). Cannulated or normal pedicle screws were placed into the vertebral bodies through a minimal access under direct vision. In a paraspinal muscle-splitting approach, mini-incision was performed to expose the root of transverse process and superior articular process. The entrance point of the pedicle was located at the junction between the 1/2 line of transverse process and the lateral border of superior articular process. A hand-held curette was used to enter the pedicle and the integrity of the pedicle was confirmed using a probe to ensure a solid tube of bone. Two suitable lengths of pedicle screws were placed into the vertebral body through the pedicle. Posteoanterior and lateral x-ray examinations were performed to check their position.

Second, normal pedicle screws were placed in all involved vertebrae at the decompression side through the paraspinal muscle-splitting approach. In an incision 2.5 cm lateral to the midline including the pedicles of two end vertebrae, the lamina, root of the transverse process, and superior articular process were exposed. Once the entry position of pedicle was identified, the pedicle screws were inserted into vertebrae as described above.

Third, unilateral MIS-TLIF was implemented *via* the expandable tubular retractor. The tubular retractor was introduced along the stepwise dilating cannulas to the facet joints and lamina through the middle point of open approach. The semi-laminae, hypertrophied superior/inferior articular processes, and ligamenta flava were removed to expose the nerve roots and dural sac for neurologic decompression (Figures 1B, 2B). After discectomy, the vertebral endplates were prepared *via* the intervertebral foramen. Sufficient autologous bone graft from the resected lamina and facets was packed into the anterior intervertebral space. A single PEEK cage containing autologous bone was inserted obliquely across the prepared intervertebral space for TLIF (Figures 1C, 2C). When there was severe spinal canal stenosis with bilateral neurologic symptoms, the expandable tubular retractor was tilted further by approximately 15° to remove the root of spinal process and the inner cortical bone of contralateral lamina for the decompression of contralateral nerve. During surgery, the nerve roots and dural sac were protected. None of the patients enrolled in the study underwent additional contralateral facet joint fusion. The decompression and fusion procedure of other spinal segments was performed as described above.

Fourth, the rods were installed over the pedicle screws. Two rods of appropriate size were contoured to maintain a normal spine curve. One rod was placed over the pedicle screws through the open approach, and the other rod was placed over the two contralateral pedicle screws of the upper and lower end vertebrae through subcutaneous soft tissues and muscles to form a “bridge”, which we termed “contralateral bridge Freehand MIPS”. On the surface of skin, a small face can be seen after this kind of MIS-TLIF, so it is also called “smile-face surgery” (Figures 1D–F, 2D–F).

#### Open TLIF

A posterior midline incision was made over the lumbar spine and the paraspinal muscles were detached from the spinous process, lamina, facet capsules and transverse processes. The pedicle screws were inserted into all involved vertebrae *via* both pedicles, followed by laminectomy and facetectomy for neurologic decompression. After discectomy was done and the endplates were prepared, autologous bone and the PEEK cage with autologous bone were inserted into intervertebral space for TLIF. Two rods were fixed over the pedicle screws (Figures 3B,C,D).

When the drainage volume was less than 20 ml/24 h, the drainage tube was pulled out. The patients were mobilized as soon as feasible after surgery. No external braces were used after surgery. After leaving the hospital, the patients were encouraged to resume their daily routine and were followed-up in the outpatient.

### Clinical follow-up

The operation time, blood loss, frequency of intraoperative fluoroscopy, length of surgical incision, hospital stay, hospitalization cost and postoperative complications were recorded.

The patient's lower back and leg pain were graded using the VAS pain rating score. ODI was used to assess the disability status preoperatively and at 5-year follow-up.

### Pre- and postprocedural imaging

All patients were evaluated before the procedure by CT and MRI imaging to determine the involved levels. x-ray examination was performed for all patients and radiographic outcomes, including disc height (DH), lumbar lordosis (LL) and segmental lordosis angle (SLA), were measured before and after surgery. DH: the vertical distance from the anterior and posterior lower endplate of upper vertebra to the upper endplate of lower vertebra is measured, and then the mean is DH; LL: the Cobb Angle between the upper endplate of L1 and the upper endplate of S1; SLA: the Cobb angle between the superior endplate of superior vertebra and the inferior endplate of inferior vertebra or the superior endplate of S1 in the surgical segment. Two experienced orthopedic surgeons who did not participate in the surgery separately evaluated the fusion status on the basis of Bridwell's posterior fusion grades (11). When there were disagreements, another radiologist was asked to assess as the final result. The cage dropped into 2 mm was considered to be subsidence according to Knox (12).

### Establish the finite element model of lumbar spine

This study was based on 3D CT data of lumbar spine before and after operation of a male patient, aged 53 years, 1.75 m in height and 72 kg in weight. Before operation, the lumbar was scanned using thin-slice CT with a slice thickness of 1 mm. Thereafter, the image was saved and exported in Dicom format, and the CT image of lumbar was modeled. The acquisition of CT data was carried out after obtaining the informed consent of patients. The finite element (FE) model of smile-face surgery and open TLIF were established by computer software respectively. Mimics 19.0 was used to build a 3D geological model in STL format for Dicom format images, and the STL file was imported into Geomagic Studio 2014 to enable the surface fitting and smoothing. The femur structure model was imported corresponding to each group into Hypermesh 14.0 software for mesh generation. The finite element model was imported into the Ansys 2021 software. The material properties of the lumbar spine's L1–S1 finite element model are listed in Table 1.

**Table 1:** Material properties of the finite element model.

Component	Material model	Young's Modulus (MPa)	Poisson's Ratio	Cross-sectional Area (mm^2^)
Cortical bone	Linear elastic	12,000	0.3	–
Cancellous bone	Linear elastic	100	0.2	–
Anterior longitudinal ligaments	Nonlinear	7.8	–	63.7
Posterior longitudinal ligaments	Nonlinear	10	–	20
Ligamentum flavum	Nonlinear	15	–	40
Facet capsule	Nonlinear	7.5	–	60
Interspinous ligament	Nonlinear	8	–	40
Supraspinous ligament	Nonlinear	10	–	30
Intertransverse ligament	Nonlinear	10	–	1.8
Screws and rods	Linear elastic	110,000	0.28	–
Polyether ether ketone cages	Linear elastic	3600	0.25	–

### Biomechanical analysis of finite element model of lumbar spine

The established finite element model was compared with that in the *in vitro* cadaver study by Yamamoto to verify the reliability of model (13). To more directly compare the stability of two kinds of fusion modes, a concentrated moment of normal physiological load (7.5 Nm) was applied. The lumbar left and right axial rotation were observed by applying a moment of normal physiological load (7.5 Nm) along the horizontal direction of L1 vertebral body upper endplate of two models. The lumbar flexion and extension were observed by applying a moment of normal physiological load (7.5 Nm) along the direction perpendicular to the L1 vertebral body upper endplate; The lateral bending was observed by applying a moment of normal physiological load (7.5 Nm) along the direction perpendicular to the L1 vertebral body upper endplate, and to observe the angle changes of model in six directions. The degree of stability of two fusion modes was compared. The changes in the stress of screws were recorded through finite element analysis, and the difference between two kinds of fusion methods were evaluated. To demonstrate the biomechanical stability of 2-level and 3-level surgery respectively, we established different models for analysis.

### Statistical analysis

Normal distributed continuous variables including age, operation time, blood loss, length of surgical incision, hospitalization cost, follow-up time, ODI, DH, LL and SLA are presented as mean ± standard deviation (SD); Categorical variables such as gender and complications are expressed as frequency or percentage; Discrete, rating variables and continuous variables, which are not normally distributed, are presented as median (Maximum- Minimum) including intraoperative fluoroscopy, drainage tube removal time, hospital stay and VAS score. *T* test is used for intergroup analysis of normal distributed continuous variables. The Mann–Whitney *U* test is used for intergroup analysis of discrete variables, rating variables, and not normally distributed continuous variables. The chi-square test is used for intergroup analysis of categorical variables. All analyses are performed using the Statistical Package for the Social Sciences (SPSS 20.0).

## Results

The characteristics of samples are detailed in Table 2. No significant differences in patients' demographic data were noted between two groups. 82 patients were retrospectively selected for this study. Based on the surgical method used, the patients were divided into 45 cases in MIS-TLIF group (34 cases with 2 segments and 11 cases with 3 segments, a total of 101 segments) and 37 cases in open TLIF group (25 cases with 2 segments and 12 cases with 3 segments, a total of 86 segments). Patients were followed up for at least five years, and the longest follow-up was 8 years.

**Table 2:** Sample characteristics of two groups.

Item	Group A	Group B	*P* value
Age (year)	59.9 ± 6.9	61.8 ± 5.6	0.761
Gender (n)			
Male	25	21	
Female	20	16	0.546
Follow-up time (months)	72.2 ± 3.2	76.5 ± 4.2	0.435
BMI	22.8 ± 3.1	23.2 ± 3.3	0.356
Intraoperative fluoroscopy	6 (5–9)	5 (5–8)	0.757
Operation time (minutes)			
2-level	123.3 ± 25.2	131.7 ± 31.2	0.546
3-level	176.6 ± 32.4	183.2 ± 36.6	0.712
Blood loss (ml)			
2-level	235.3 ± 20.1	411.5 ± 31.2	<0.01*
3-level	312.4 ± 30.6	530.2 ± 45.3	<0.01*
Surgical incision (cm)			
2-level	4.2 ± 1.7	8.3 ± 2.4	<0.01*
3-level	6.1 ± 2.2	12.3 ± 3.6	<0.01*
Hospitalization cost (¥)			
2-level	62,435 ± 3875	68,634 ± 4722	<0.01*
3-level	72,642 ± 4189	80,132 ± 4375	<0.01*
Hospital stay (days)	6 (4–8)	8 (6–15)	0.031*
Removal of drainage tube (days)	3 (2–4)	4 (3–10)	0.783
Complications			
Neurological injury	0	0	
Dural tear	0	2 (2.3%)	
Cage sedimentation	0	0	
Urinary tract infection	0	2 (5.4%)	
Wound infection	0	1 (2.7%)	
Severe low back pain	0	4 (10.8%)	

*P* value is used for comparison between two groups.

* *P* < 0.05, statistically significant.

### Clinical efficacy evaluation

Smile-face surgery and open TLIF were successfully performed for all cases. The blood loss, length of operative incision, and hospital stay in MIS-TLIF group were significantly less than those in open TLIF group. There was no significant difference in the frequency of intraoperative fluoroscopy and operation time between two groups (Table 2). The VAS of low back in MIS-TLF group was significantly lower than that in open TLIF group immediately and 3 months after surgery, and no significant difference was observed 1 year, 2 years and 5 years after surgery. There was no significant difference in the postoperative VAS of leg pain and the ODI score at 5-year follow-up between MIS-TLIF group and open MIS-TLIF group (Table 3). The complication rate of open TLIF was higher than that of MIS-TLIF (24.32% vs. 0%, *P* < 0.01) (Table 2).

**Table 3:** VAS of low back, leg and ODI in two groups.

	Group	Preoperation	3-month	6-month	5-year
VAS of low back	Group A	6 (3–8)	3 (2–8)^a^^*^	3 (2–6)^a^	2 (2–3)^a^
	Group B	6 (2–8)	5 (2–8)^a^	4 (2–6)^a^	2 (2–4)^abc^
	*P* value	0.65	<0.05	0.58	0.45
VAS of leg	Group A	7 (5–9)	2 (0–2)^a^	1 (0–1)^a^	1 (0–1)^a^
	Group B	7 (5–9)	2 (0–2)^a^	1 (0–)^a^	1 (0–1)^a^
	*P* value	0.65	0.54	0.54	0.69
ODI (%)	Group A	62.3 ± 10.1	–	–	4.8 ± 2.4^a^
	Group B	58.4 ± 10.3	–	–	5.4 ± 2.9^a^
	*P* value	0.65	–	–	0.63

ODI, oswestry disability index; VAS, visual analogue scale.

* *P* < 0.05, comparison between two groups.

^a^ *P* < 0.05, compared with preoperatively.

^b^ *P* < 0.05, compared with 3-month follow-up.

^c^ *P* < 0.05, compared with 6-month follow-up.

### Radiographic outcomes

Postoperative x-ray and CT confirmed that the position of cages and screws was good and the neurologic decompression was complete (Figures 1E–G, 2E–G, 3B,C,E). DH, LL and SLA significantly improved after surgery (*P* < 0.05). There was no significant difference between two groups in the DH, LL and SLA preoperatively, immediately, 3 months, 6 months, 1 year, 2 years and 5 years postoperatively (Table 4). At 5-year follow-up, 63 segments with Grade I fusion, 37 segments with Grade II fusion and 1 segments with Grade III fusion were observed in MIS-TLIF group (Figures 1H, 2H); In open TLIF group, 51 cases with Grade I fusion, 36 cases with Grade II fusion and 2 cases with Grade III fusion in were observed (Figure 3E).

**Table 4:** Dh, LL and SLA of two groups.

	Group	Preoperatively	Immediately	5-year
DH	Group A	8.8 ± 2.4	13.0 ± 2.9^a^	12.1 ± 3.1^a^
	Group B	8.4 ± 2.6	12.6 ± 2.4^a^	11.9 ± 2.9^a^
	*P* value	0.65	0.55	0.52
LL	Group A	40.8 ± 4.9	47.2 ± 5.1^a^	45.7 ± 5.7^a^
	Group B	42.1 ± 4.6	55.4 ± 4.9^a^	46.4 ± 5.8^a^
	*P* value	0.73	0.65	0.61
SLA	Group A	16.9 ± 2.8	23.1 ± 5.2^a^	21.1 ± 3.2^a^
	Group B	17.4 ± 2.2	25.4 ± 5.6^a^	23.4 ± 3.6^a^
	*P* value	0.76	0.65	0.58

DH, disc height; LL, lumbar lordosis; SLA, segmental lordosis angle.

^a^ *P* < 0.05, compared with preoperatively.

### Verification of finite element model

**Figure 4:**
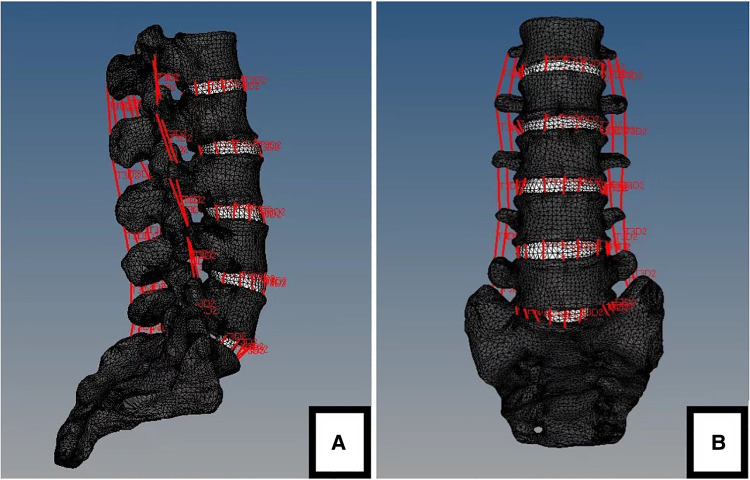
The finite element model of stable lumbar (L1-S1). (**A**) lateral view. (**B**) anteroposterior view.

A moment of physiological load of 7.5 Nm was applied to the L1 in all directions, and the angles of movement in the directions of flexion, extension, lateral bending, and axial rotation were compared. As measured, the range of motion (ROM) of finite element model under the six directions was similar to that of *in vitro* model, and the differences in the results were acceptable considering the individual differences in the models themselves. Therefore, the established finite element model can accurately simulate the biological structure of lumbar spine (Table 5, Figure 4).

**Table 5:** Comparison of range of motion between the finite element model and the cadaveric study by Yamamoto et al.

Moment	Level	Yamamoto's research	Model
Flexion (°)	L1-2	4.2 ± 0.4	4.4
	L2-3	5.4 ± 0.3	5.6
	L3-4	6.1 ± 0.6	6.4
	L4-5	7.1 ± 0.6	7.4
	L5-S1	7.0 ± 0.6	7.3
Extension (°)	L1-2	2.8 ± 0.3	3.0
	L2-3	3.3 ± 0.3	3.4
	L3-4	2.3 ± 0.2	2.3
	L4-5	4.0 ± 0.5	4.3
	L5-S1	4.8 ± 0.6	5.0
Lateral bending (°)	L1-2	3.7 ± 0.1	3.7
	L2-3	5.1 ± 0.4	5.3
	L3-4	4.4 ± 0.3	4.5
	L4-5	4.3 ± 0.4	4.4
	L5-S1	3.9 ± 0.3	4.0
Axial rotation (°)	L1-2	1.7 ± 0.4	1.8
	L2-3	1.4 ± 0.3	1.5
	L3-4	2.0 ± 0.3	2.0
	L4-5	1.4 ± 0.2	1.4
	L5-S1	1.1 ± 0.2	1.2

### Maximum Von mises stress of lumbar interbody fusion model

**Figure 5:**
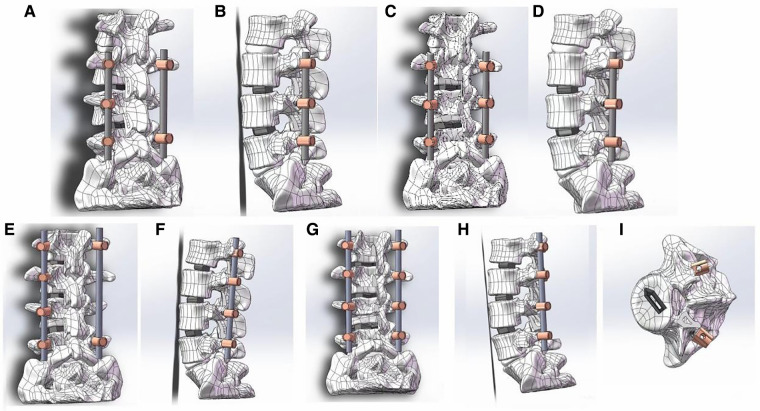
The procedure of two kinds of TLIF simulation and establishment of postoperative FE model. (**A**) Schematic diagram of 2-level smile-face surgery: positive view; (**B**) Schematic diagram of 2-level smile-face surgery: lateral view; (**C**) Schematic diagram of 2-level open TLIF surgery: positive view; (**D**) Schematic diagram of 2-level open TLIF surgery: lateral view; (**E**) Schematic diagram of 3-level smile-face surgery: positive view; (**F**) Schematic diagram of 3-level smile-face surgery: lateral view; (**G**) Schematic diagram of 3-level open TLIF surgery: positive view; (**H**) Schematic diagram of 3-level open TLIF surgery: lateral view; (**I**) vertical view of bullet cage.

**Figure 6:**
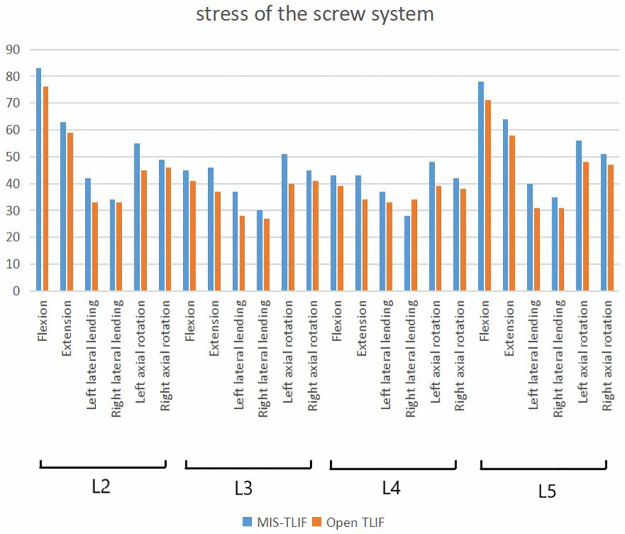
The stress value of screw systems in two kinds of 3-level model under different conditions.

**Figure 7:**
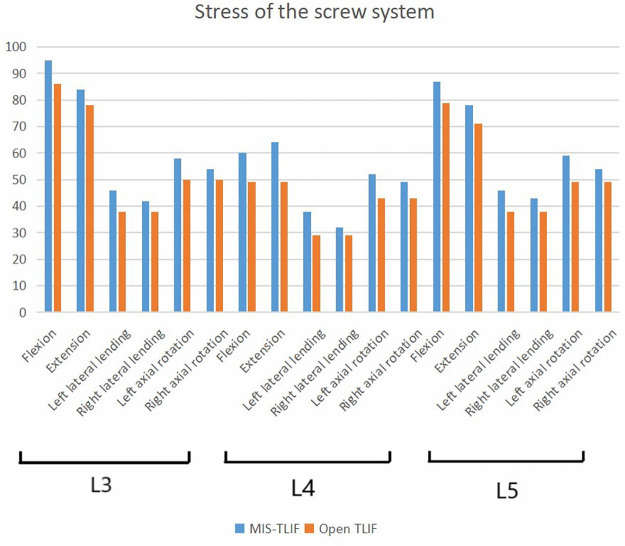
The stress value of screw systems in two kinds of 2-level model under different conditions.

We next analyzed the stress of two kinds of fusion (Figure 5): In the 3-level model, the von Mises stress of screw of smile-face surgery ranged in 83 MPa in L2, in 45 MPa L3, 43 MPa in L4 and 78 MPa in L5 and the open TLIF ranged in 76 MPa in L2, in 41 MPa L3, in 39 MPa L4, in 71 MPa L5 in the flexion direction; The von Mises stress of screw of MIS-TLIF ranged in 63 MPa in L2, 46 MPa in L3, 43 MPa in L4 and 64 MPa in L5 and the open TLIF ranged in 59 MPa in L2, 37 MPa in L3, 34 MPa in L4, 34 MPa in L5 in the extension direction; The von Mises stress of screw of MIS-TLIF ranged in 42 MPa in L2, 37 MPa in L3, 37 MPa in L4 and 40 MPa in L5 and the open TLIF ranged 33 MPa in L2, 28 MPa in L3, 33 MPa in L4, 31 MPa in L5 in the left lateral bending direction; The von Mises stress of screw of MIS-TLIF ranged in 34 MPa in L2, 30 MPa in L3, 28 MPa in L4 and 35 MPa in L5 and the open TLIF ranged 33 MPa in L2, 27 MPa in L3, 34 MPa in L4, 31 MPa in L5 in the right lateral bending direction; The von Mises stress of screw of MIS-TLIF ranged in 55 MPa in L2, 51 MPa in L3, 48 MPa in L4 and 56 MPa in L5 and the open TLIF ranged in 45 MPa in L2, 40 MPa in L3, 39 MPa in L4, 48 MPa in L5 in the left axial rotation direction; The von Mises stress of screw of MIS-TLIF ranged in 49 MPa in L2, 45 MPa in L3, 42 MPa in L4 and 51 MPa in L5 and the open TLIF ranged in 46 MPa in L2, 41 MPa in L3, 38 MPa in L4, 47 MPa in L5 in the right axial rotation direction (Figure 6). In the 2-level model, the von Mises stress of screw of smile-face surgery ranged in 95 MPa in L3, 60 MPa in L4 and 87 MPa in L5 and the open TLIF ranged in 86 MPa in L3, 49 MPa in L4, 79 MPa in L5 in the flexion direction; The von Mises stress of screw of MIS-TLIF ranged in 84 MPa in L3, 64 MPa in L4 and 78 MPa in L5 and the open TLIF ranged in 78 MPa in L3, 49 MPa in L4, 71 MPa in L5 in the extension direction; The von Mises stress of screw of MIS-TLIF ranged in 46 MPa in L3, 38 MPa in L4 and 46 MPa in L5 and the open TLIF ranged in 38 MPa in L3, 29 MPa in L4, 38 MPa in L5 in the left lateral bending direction; The von Mises stress of screw of MIS-TLIF ranged in 42 MPa in L3, 32 MPa in L4 and 43 MPa in L5 and the open TLIF ranged in 38 MPa in L3, 29 MPa in L4, 38 MPa in L5 in the right lateral bending direction; The von Mises stress of screw of MIS-TLIF ranged in 58 MPa in L3, 52 MPa in L4 and 59 MPa in L5 and the open TLIF ranged in 50 MPa in L3, 43 MPa in L4, 49 MPa in L5 in the left axial rotation direction; The von Mises stress of screw of MIS-TLIF ranged in 54 MPa in L3, 49 MPa in L4 and 54 MPa in L5 and the open TLIF ranged in 50 MPa in L3, 43 MPa in L4, 49 MPa in L5 in the right axial rotation direction. When the number of segments increased, the pressure difference between MIS-TLIF and open TLIF increased (Figure 7 and Supplementary table 1)

### Changes in ROM according to lumbar interbody fusion model

The changes in the angles of axial rotation, flexion-extension, and lateral bending of two model under the same load were compared, and all two models had a stable structure under normal physiological load. Under the normal physiological load condition, the angles of movement in the directions of flexion, extension, lateral bending, and axial rotation of two kinds of fusion were then recorded. In the 3-level model, the overall angle of MIS-TLIF is 0.39 and open TLIF is 0.32 in the flexion direction; The overall angle of MIS-TLIF is 0.36 and open TLIF is 0.25 in the extension direction; The overall angle of MIS-TLIF is 0.17 and open TLIF is 0.14 in the lateral bending direction; The overall angle of MIS-TLIF is 0.21 and open TLIF is 0.2 in the axial rotation direction (Table 6). In the 2-level model, the overall angle of MIS-TLIF is 0.23 and open TLIF is 0.18 in the flexion direction; The overall angle of MIS-TLIF is 0.24 and open TLIF is 0.2 in the extension direction; The overall angle of MIS-TLIF is 0.18 and open TLIF is 0.16 in the lateral bending direction; The overall angle of MIS-TLIF is 0.19 and open TLIF is 0.17 in the axial rotation direction (Table 7).

**Table 6:** Range of motion in 3-level model.

Moment	Level	MIS-TLIF	Open TLIF
Flexion (°)	L2-3	0.13	0.10
	L3-4	0.11	0.09
	L4-5	0.15	0.13
Extension (°)	L2-3	0.13	0.06
	L3-4	0.11	0.08
	L4-5	0.12	0.11
Lateral bending (°)	L2-3	0.17	0.14
	L3-4	0.09	0.08
	L4-5	0.05	0.04
Axial rotation (°)	L2-3	0.19	0.18
	L3-4	0.13	0.11
	L4-5	0.21	0.20

**Table 7:** Range of motion in 2-level model.

Moment	Level	MIS-TLIF	Open TLIF
Flexion (°)	L3-4	0.11	0.09
	L4-5	0.12	0.09
Extension (°)	L3-4	0.11	0.09
	L4-5	0.13	0.11
Lateral bending (°)	L3-4	0.12	0.11
	L4-5	0.06	0.05
Axial rotation (°)	L3-4	0.17	0.11
	L4-5	0.19	0.17

## Discussion

Lumbar fusion surgery is an effective surgical procedure for the treatment of spinal degenerative disorders (1). Since the introduction of PLIF by Cloward (2, 3) in 1952, new techniques have been developed to accomplish lumbar interbody fusion. In 1982, TLIF, an alternative to PLIF, was introduced for the treatment of spinal degenerative diseases that necessitated interbody fusions (4). TLIF provides a more lateral surgical approach for the space of lumbar disc to reduce the retraction of dural sac and nerve root, and to avoid the postoperative midline scar that hinders the identification of neural structures in revision patients and confers the lowest post-operative disability. The clinical outcomes of open TLIF or PLIF surgery have been good, but several studies reported that the muscle damage from subperiosteal curettage affected clinical prognosis adversely (14, 15). Wiltse (16) described a paraspinal sacrospinalis muscle-splitting approach to the lumbar spine, which reduced bleeding and provided a direct route to the transverse processes and pedicle. Compared with traditional midline incisions, this technique was thought to reduce postoperative pain and avoid rupture of the supraspinous ligament and interspinous ligament. Since Foley (17, 18) proposed the Wiltse approach for MIS-TLIF, many scholars have reported its significant advantages over open PLIF and TLIF (19–21).

Advances in MIS-TLIF have led to two predominant approaches: mini-open with expandable tubular retractor through a bilateral Wiltse approach, and minimally invasive surgery using one non-expandable or expandable tubular retractor and bilateral percutaneous screw placements, which is performed for fusions of 1 or 2 segments (22). Unilateral pedicle screw fixation for MIS-TLIF is widely used to treat single-level LDD (23). Currently there is no optimal minimally invasive treatment strategy for multi-segmental LDDs. Unilateral pedicle screw fixation or plus transarticular screws might not supply enough biomechanical stability for MIS-TLIF in multi-segmental fusion. Contralateral percutaneous pedicle screw fixation (PPS) guided by C-arm for all involved vertebrae could increase the incision number, radiological exposure, operative duration and difficulty of rod installation. Therefore, we designed a contralateral bridge Freehand MIPS combined with unilateral MIS-TLIF for bilateral neurological decompression to treat multi-segmental (2- or 3-level) LDDs. The results showed that the VAS score of leg pain was significantly reduced during follow-up (*P < *0.01) and the ODI was significantly reduced 5 years after surgery (*P < *0.01) compared with preoperative values, which is similar to the clinical outcomes of open TLIF.

During the unilateral MIS-TLIF of this study, the normal pedicle screws are placed at the decompression side into all involved vertebrae through a paramedian muscle-splitting approach, which can provide a direct access to pedicles resulting in the incision shortened. In open TLIF, a longer midline incision is needed for the insertion of pedicle screws (4.2 ± 1.7 cm vs. 8.3 ± 2.4 cm in 2-level, 6.1 ± 2.2 cm vs. 12.3 ± 3.6 cm in 3-level). In MIS-TLIF group, the two pedicle screws are needed to be inserted into the upper and lower end vertebrae when performing contralateral bridge Freehand MIPS, and the rods are placed on the pedicle screws through subcutaneous soft tissues and muscles. Freehand MIPS is used to insert the pedicle screws into the vertebrae through Wiltse approach in a mini-incision under direct vision (5–10). Both Freehand MIPS and unilateral MIS-TLIF could protect the attachments of paraspinous musculature to spinal processes and the natural posterior tension band including the supraspinous and interspinous ligaments (17, 18). The use of a tubular retraction system in unilateral MIS-TLIF preserves healthy muscle tissue and further decreases damage to the ipsilateral paraspinous musculature (24). Although this procedure requires the removal of a complete unilateral facet joint, it is possible to obtain the decompression of bilateral nerves and preserve the integrity of contralateral facet joints. All these can help reducing the intraoperative bleeding and postoperative pain. In our research, the blood loss of MIS-TLIF (235.3 ± 20.1 in 2-level, 312.4 ± 30.6 in 3-level) was significantly lower than that in open TLIF (411.5 ± 31.2 in 2-level, 530.2 ± 45.3 in 3-level). MIS-TLIF group showed earlier drainage removal and shorter hospital stay than open TLIF. In some patients of open TLIF, the delayed time to pull out the drainage tube and catheter leaded to urinary tract infection. The VAS of low back in MIS-TLIF group was significantly lower than that in open TLIF group within 3 months follow-up. Some patients treated by open TLIF had intractable low back pain.

In MIS-TLIF, the unilateral facetectomy provides a complete exposed field of far-lateral aspect of intervertebral disc space, so that little retraction of thecal sac and/or nerve roots is required when preparing the intervertebral disc space and placing the cage (25). The retraction of neural elements is unilateral and minimal, signiﬁcantly decreasing the risk of neurologic injury and dura tear caused by traction. In addition, pedicle screw fixation has some risks because it can cause nerve injury (26). In Freehand MIPS, the pedicle must be carefully probed in all four quadrants to ensure that a solid tube of bone exists and that violation into the inferiorly neuroforamen or into the spinal canal does not occur before the pedicle screws are implanted into the vertebrae under direct vision. This measure is taken to guarantee the safety of surgery and avoid neurologic deficits without dependence on fluoroscopy. In this study there were no serious neurologic complications, and postoperative radiography and scanning images showed that the screws and cages were correctly positioned, which confirmed the safety of smile-face surgery. Compared with percutaneous pedicle screw fixation (PPS), Freehand MIPS requires a surgical incision of similar size, but has some advantages including less intraoperative fluoroscopy monitoring and easier manipulation during surgery. Either cannulated pedicle screws or common pedicle screws can be used in Freehand MIPS, whereas only cannulated screws are used for PPS. Bridge fixation of Freehand MIPS with two pedicle screws for two end vertebrae allows the rapid installation of pedicle screws and easy implantation of rod compared with contralateral pedicle screw fixation for all involved vertebrae.

According to the follow-up result of imaging, there was no significant difference in the postoperative DH, SLA, and LLA between MIS-TLIF and open TLIF. Fusion rate was 99% (100/101) in MIS-TLIF and 97% (84/86) in open TLIF at 5-year follow-up and there was no instrumentation failure such as loosening or fracture of screws and rods. The fusion rate of MIS-TLIF is not less than that in open TLIF. This finding verified that sufficient biomechanical stability was achieved by contralateral bridge Freehand MIPS combined with unilateral MIS-TLIF. With simulation, veracity and repeatability, the finite element analysis has been viewed as a reliable approach for evaluating the biomechanical behavior of different internal fixation system, which is comparable with traditional cadaver research. We can get the same results as the cadaver model by simulating the biomechanical experiment with finite element analysis. Here, we carry out a FE analysis and a series of model measurement researches to unveil the biomechanical difference between two fusion methods. In TLIF pedicle screws fixation is the main force for stable lumbar reconstruction, but there are some postoperative problems mainly including loosening and breakage of screws. For evaluating the stress of pedicle screws fixation system, flexion, extension, left axial rotation and right axial rotation are important working conditions that cause stress concentration (27). The difference in the position and number of pedicle screws is the main reason for the difference in stress distribution (28, 29). Different number and direction will change the distribution of stress of pedicle screws and rods, resulting in screw fracture and loosening. The stress in pedicle screws tends to be concentrated on the end of thread at the tail of screw, so screw breakage often occurs here (30). Flexion and extension after posterior lumbar surgery remain the most dangerous condition (31). In the model of flexion and extension of this study, bridge fixation did not significantly increase the stress of pedicle screws compared with open-TLIF. During axial rotation, the upper and lower screw stress of bridge fixation increased more than those of open-TLIF. The reason may be that during axial rotation, the fully fixed side was taken as the center of rotation, and the incomplete fixed side needed to bear more torque. But axial rotation is not the main direction of motion in the lumbar spine. What's more, the stress distribution on the screw during axial rotation and lateral bending is also significantly less than that during lumbar flexion and extension, so the increase in stress during axial rotation does not significantly affect the overall fixation. The finite element analysis of this study supports that there is no significant difference in biomechanical stability between two kinds of fusion.

Various factors may decrease the direct and indirect costs of smile-face surgery for multi-level LDDs. Compared with contralateral pedicle screw fixation for all involved levels, bridge fixation reduces the number of pedicle screws used and implantation fees. Less blood loss can avoid the need for blood transfusion during the procedure, which might also decrease the cost of patient (32). The less postoperative pain might also lower the cost of analgesics. This type of MIS-TLIF intervention may result in a shorter length of hospitalization and lower complication rates (33), which might reduce the utilization of hospital resources. In addition, the faster recovery rate suggests that MIS-TLIF patients do not need long-time inpatient rehabilitation after surgery, further reducing the overall cost. Finally, a rapid return to work and productivity means lower indirect costs to the patient and society.

## Limitation

1. Smile-face surgery is a technically demanding procedure that has a learning curve. Good outcomes can be achieved if the surgeons have prior experience in open TLIF. As the surgical technique matures, the complication rate significantly decreases.

2. This study was a retrospective analysis, so there might be some bias in the selection of cases. When some patients had very severe spinal stenosis on imaging, the surgeon would more likely to use open TLIF. Due to severe spinal stenosis resulting in adhesion of dura and ligamentum flavum, lamina, the dura was easy to tear during the removal of lamina.

3. Normal physiological loads were applied to the model without destructive or excessive loads in the finite element analysis. The results of this study are not applicable if the patient has undergone excessive exercise (overload) or trauma (destructive load).

## Conclusions

The contralateral bridge Freehand MIPS combined with unilateral MIS-TLIF (smile-face surgery) has advantages over open TLIF including smaller aggression, less blood loss, and lower cost, indicating that it is a good choice of treatment for multi-segmental LDDs. Both methods can achieve good biomechanical stability. This has a certain reference value for currently popular technique: multi-segmental percutaneous endoscopic TLIF (PE-TLIF).

## Data Availability

The original contributions presented in the study are included in the article/**Supplementary Material**, further inquiries can be directed to the corresponding author/s.
