# Supplementary material for: Contralateral bridge fixation of freehand minimally invasive pedicle screws combined with unilateral MIS-TLIF vs. open TLIF in the treatment of multi-segmental lumbar degenerative diseases: A five years retrospective study and finite element analysis
Source: Front Surg. 2022 Nov 2;9:1049260. doi: 10.3389/fsurg.2022.1049260 (PMC9666694; doi:10.3389/fsurg.2022.1049260)
Supplement: Supplementary file 1 [file Table1.doc]

Maximum pressure on the screw in 3-level model.

| Moment | Level | MIS-TLIF | open TLIF |
| --- | --- | --- | --- |
| Flexion(MPa) | L2 | 83 | 76 |
|  | L3 | 45 | 41 |
|  | L4 | 43 | 39 |
|  | L5 | 78 | 71 |
| Extension(MPa ) | L2 | 63 | 59 |
|  | L3 | 46 | 37 |
|  | L4 | 43 | 34 |
|  | L5 | 64 | 58 |
| Left lateral bending(MPa ) | L2 | 42 | 33 |
|  | L3 | 37 | 28 |
|  | L4 | 37 | 33 |
|  | L5 | 40 | 31 |
| Right lateral bending(MPa ) | L2 | 34 | 33 |
|  | L3 | 30 | 27 |
|  | L4 | 28 | 34 |
|  | L5 | 35 | 31 |
| Left axial rotation(MPa ) | L2 | 55 | 45 |
|  | L3 | 51 | 40 |
|  | L4 | 48 | 39 |
|  | L5 | 56 | 48 |
| Right axial rotation(MPa ) | L2 | 49 | 46 |
|  | L3 | 45 | 41 |
|  | L4 | 42 | 38 |
|  | L5 | 51 | 47 |

Maximum pressure on the screw in 2-level model.

| Moment | Level | MIS-TLIF | open TLIF |
| --- | --- | --- | --- |
| Flexion(MPa ) | L3 | 95 | 86 |
|  | L4 | 60 | 49 |
|  | L5 | 87 | 79 |
| Extension(MPa ) | L3 | 84 | 78 |
|  | L4 | 64 | 49 |
|  | L5 | 78 | 71 |
| Left lateral bending(MPa ) | L3 | 46 | 38 |
|  | L4 | 38 | 29 |
|  | L5 | 46 | 38 |
| Right lateral bending(MPa ) | L3 | 42 | 38 |
|  | L4 | 32 | 29 |
|  | L5 | 43 | 38 |
| Left axial rotation(MPa ) | L3 | 58 | 50 |
|  | L4 | 52 | 43 |
|  | L5 | 59 | 49 |
| Right axial rotation(MPa ) | L3 | 54 | 50 |
|  | L4 | 49 | 43 |
|  | L5 | 54 | 49 |
